# Signal Balancing by the CetABC and CetZ Chemoreceptors Controls Energy Taxis in *Campylobacter jejuni*

**DOI:** 10.1371/journal.pone.0054390

**Published:** 2013-01-29

**Authors:** Mark Reuter, Arnoud H. M. van Vliet

**Affiliations:** Institute of Food Research, Gut Health and Food Safety Programme, Norwich Research Park, Norwich, United Kingdom; Charité-University Medicine Berlin, Germany

## Abstract

The coupling of environmental sensing to flagella-mediated directed motility allows bacteria to move to optimum environments for growth and survival, either by sensing external stimuli (chemotaxis) or monitoring internal metabolic status (energy taxis). Sensing is mediated by transducer-like proteins (Tlp), either located in the membrane or in the cytoplasm, which commonly influence motility via the CheA-CheY chemotaxis pathway. In this study we have investigated the role of PAS-domain-containing intracellular Tlp-sensors in energy taxis of the food-borne pathogen *Campylobacter jejuni*, using plate- and tube-based assays utilising the conversion of the redox indicator dyes triphenyl tetrazolium chloride (TTC) and resazurin. Inactivation of the genes encoding the *Campylobacter* Energy Taxis system (CetA (Tlp9) and CetB (Aer2)) in *C. jejuni* strain NCTC 11168 resulted in reduced taxis. Inactivation of the *cj1191c* gene, encoding the CetB homolog CetC (Aer1), did not affect taxis *per se*, but the *cetC* gene complemented a *cetB* mutant *in trans*, indicating that CetC can form a functional signal transduction complex with CetA in the absence of CetB. Inactivation of both CetB and CetC resulted in greatly reduced taxis confirming the role of CetC in energy taxis. Inactivation of the *cj1110c* gene, encoding Tlp8 (CetZ), a cytoplasmic sensor with two PAS-domains, resulted in increased taxis, a phenotype opposite to that of CetAB. Inactivation of the *cheA* gene resulted in the same overall phenotype as the *cetAB* mutant in both wild-type and *cetZ* backgrounds, suggesting that both systems use the CheA system for signal transduction. Absence of both CetAB and CetZ resulted in the *cetAB* taxis phenotype, suggesting that CetZ is subordinate to CetAB. In conclusion, we present evidence that *C. jejuni* balances the input from two counteracting PAS-domain-containing sensory systems to position itself for optimal usage of energy resources.

## Introduction

Bacteria are well adapted to survive in various niches, but long-term survival will often require transit through stressful environments. Consequently, bacteria have adopted a variety of mechanisms to sense and respond to these environmental stresses. Long-term genetic adaptations are possible by horizontal gene transfer, genomic deletions or reorganisation, or through random mutation and selection [1]. Relatively short-term adaptations are possible by sensing environmental changes and modulating gene expression, as typified by the two-component regulatory systems [2]. However, the most rapid adaptation a motile bacterium can make is to use taxis-controlled motility to move away from stress or move towards a more favourable locale [3].

Bacterial taxis encompasses sensing a specific environmental or internal signal, which triggers a signal transduction cascade, resulting in changing the direction of flagella rotation to affect a change in swimming direction. The paradigm of bacterial taxis is the chemotaxis system characterised in *Escherichia coli* [4]. Specifically, activation of a signal receptor results in auto-phosphorylation of CheA, with subsequent phosphotransfer to CheY. Phospho-CheY interacts with the flagella motor proteins, which alters the direction of flagella rotation from counter-clockwise (swimming phenotype) to clockwise rotation (tumbling phenotype). Accordingly, the population of bacteria change direction, albeit in a somewhat stochastic manner [5].

While the CheA-CheY phospho-relay pathway serves as the ‘master’ switch controlling taxis, the task of sensing a diverse set of environmental signals falls to a group of sensing proteins termed either methyl-accepting chemotaxis proteins (MCPs) [6] or transducer-like proteins (Tlps) [7]. Such sensing systems give bacteria the ability to exhibit taxis responses to gradients of small molecules including nutrients (chemotaxis), pH (pH taxis), light (phototaxis), magnetic fields (magnetotaxis), and intracellular energy status (aerotaxis, redox taxis, or energy taxis) [3]. Energy taxis allows bacteria to swim to environments that are optimal for energy generation, whether that means sensing an optimal oxygen concentration, appropriate electron acceptors, light, or redox-active compounds [8], [9]. For example, the *E. coli* aerotaxis sensor Aer mediates taxis to optimum oxygen concentrations through direct interaction with the respiratory chain [10], whereas the *Azospirillum brasilense* AerC protein is a redox sensor that mediates taxis to microaerobic conditions optimal for nitrogen fixation [11]. One of the protein domains associated with energy taxis is the PAS (Per-ARNT-Sim) domain, which is the most common sensing domain present in bacterial MCP proteins [6]. The PAS domain is found in all kingdoms of life, and has been described to be involved in sensing diverse stimuli including light, oxygen, redox potential, small metabolites, and proton-motive force [12], and is often involved in energy taxis [8], [9].

Many bacterial pathogens require directed motility for infection and accordingly possess functional taxis systems; the human pathogen *Campylobacter jejuni* is no exception. *C. jejuni* is leading cause of foodborne illness in the developed world (over 60,000 confirmed cases annually in the UK [13]), and is most often associated with the consumption or handling of under-cooked poultry products [14]. Despite its relatively small size, the *C. jejuni* genome encodes ten proteins containing MCP-domains [7], which is considerably more than the five, including Aer, found in *E. coli*. Indeed, based on the Pfam database [15], the MCP domain is the fifth most prevalent domain in *C. jejuni*. This is similar to the average number of MCPs predicted in bacterial genomes, but considerably more than the average number found in pathogens and commensals [6]. For *C. jejuni*, formate and asparate sensors have been identified [16], [17], as well as a proposed energy taxis system, CetAB (*Campylobacter* energy taxis proteins A and B) [18]. The CetAB system consists of two co-transcribed open reading frames [19] encoding a discrete membrane-bound MCP protein and cytoplasmic PAS-domain protein (CetA (Tlp9) and CetB (Aer2), respectively), which is atypical for such sensors, as these usually consist of a single protein with a modular architecture containing a C-terminal MCP domain, and an N-terminal ligand binding domain (LBR) [6].

Inactivation of *cetA* and *cetB* in *C. jejuni* strain 81–176 resulted in decreased motility in defined agar plates supplemented with pyruvate and fumarate [18]. The CetA and CetB proteins were further shown to directly interact, with CetB being associated with membrane-bound CetA [19]. Based on the structure and genetic organization of the *cetAB* genes, these have been proposed to constitute a new class of bipartite energy taxis systems, with a number of such systems found outside the genus *Campylobacter* [20].

In addition to *cetAB*, the *C. jejuni* genome contains two other genes encoding PAS-domain containing proteins implicated in taxis: *cj1110c* (*tlp8*), which encodes an MCP protein which contains two predicted PAS domains, and *cj1191c* (*aer1*), which encodes a homolog of CetB and contains a single predicted PAS domain. In this study, we have investigated the role of the PAS- and MCP-domain genes *cetAB*, *cj1191c* and *cj1110c* in *C. jejuni* energy taxis. We have used genetic mutation and complementation analyses, swarm plate assays, and a newly developed tube taxis assay utilising redox dyes to show that the CetAB system is required for efficient energy taxis in a redox gradient. We demonstrate that Cj1191c is involved in energy taxis, and further show that Cj1110c is a sensor involved in a CheA-dependent redox taxis response that is opposed, but subordinate to the response mediated by the CetAB system. These results suggest that *Campylobacter* has the ability to balance redox and energy status signals, via PAS-domain linked signal transduction, to mediate taxis to an optimal environment.

## Materials and Methods

### *C. jejuni* strains and growth conditions

*Campylobacter jejuni* strain NCTC 11168 and its isogenic mutants (Table 1)were routinely cultured in a MACS-MG-1000 controlled atmosphere cabinet (Don Whitley Scientific) under microaerobic conditions (85% N_2_, 5% O_2_, 10% CO_2_) at 37°C [21]. For growth on plates, strains were either grown on Brucella agar, blood plates (Blood Agar Base 2 (BAB), 1% yeast extract, 5% horse blood (Oxoid)), or BAB with Skirrow supplements (10 µg ml^−1^ vancomycin, 5 µg ml^−1^ trimethoprim, 2.5 IU polymyxin-B). Broth culture was carried out in Brucella broth (Becton Dickinson). An Innova 4230 (New Brunswick Scientific) incubator was used for aerobic culture at 37°C and a Sanyo MCO-20 AIC incubator was used for culture in 10% CO_2_ in air at 37°C.

**Table 1:** Bacterial strains used in this study.

Strain	Description^a^
***E. coli*** ** strains**	
Top10	General cloning strain
***C. jejuni*** ** strains**	
NCTC 11168	Wild-type *C. jejuni* [46]
11168 Δ*cetA*	NCTC 11168 *cj1190c::kan^R^*
11168 Δ*cetB*	NCTC 11168 *cj1189c::kan^R^*
11168 Δ*cetAB*	NCTC 11168 (*cj1189-90c*)*::kan^R^*
11168 Δ*cetC*	NCTC 11168 *cj1191c::kan^R^*
11168 Δ*cetZ*	NCTC 11168 *cj1110c::cat^R^*
11168 Δ*cheA*	NCTC 11168 *cj0284c::kan^R^*
11168 Δ*cheA* Δ*cetZ*	NCTC 11168 *cj0284c::kan^R^ cj1110c::cat^R^*
11168 Δ*flaAB*	NCTC 11168 (*cj1338-39c*)*::kan^R^*
11168 Δ*cetA* Δ*cetZ*	NCTC 11168 *cj1190c::kan^R^ cj1110c::cat^R^*
11168 Δ*cetB* Δ*cetZ*	NCTC 11168 *cj1189c::kan^R^ cj1110c::cat^R^*
11168 Δ*cetAB* Δ*cetZ*	NCTC 11168 (*cj1189-90c*)*::kan^R^ cj1110c::cat^R^*
11168 Δ*cetB* Δ*cetC*	NCTC 11168 *cj1189c::kan^R^ cj1191c::cat^R^*
11168 Δ*cetA::cetA*fdxApr*	NCTC 11168 *cj1190c::kan^R^ cj0046*::*cetA*fdxApr* *cat^R^*
11168 Δ*cetA::cetA* ^cetApr^ *	NCTC 11168 *cj1190c::kan^R^ cj0046*::*cetA* ^cetApr^ * *cat^R^*
11168 Δ*cetA::cetAB*fdxApr*	NCTC 11168 *cj1190c::kan^R^ cj0046*::*cetAB*fdxApr* *cat^R^*
11168 Δ*cetB::cetAB*fdxApr*	NCTC 11168 *cj1189c::kan^R^ cj0046*::*cetAB*fdxApr* *cat^R^*
11168 Δ*cetAB::cetAB*fdxApr*	NCTC 11168 (*cj1189-90c*)*::kan^R^ cj0046*::*cetAB*fdxApr* *cat^R^*
11168 Δ*cetB::cetB* *	NCTC 11168 *cj1189c::kan^R^ cj0046*::*cetB*fdxApr* *cat^R^*
11168 Δ*cetB::cetC* *	NCTC 11168 *cj1189c::kan^R^ cj0046*::*cj1191c*fdxApr* *cat^R^*
11168 Δ*cetAB::cetA-cetB* *	NCTC 11168 (*cj1189-90c*)*::kan^R^ cj0046*::*cetA-cetB*fdxApr* *cat^R^*
11168 Δ*cetAB::cetA-cetC* *	NCTC 11168 (*cj1189-90c*)*::kan^R^ cj0046*::*cetA*-*cj1191c*fdxApr* *cat^R^*
11168 Δ*cetZ::cetZ* *	NCTC 11168 *cj1110c::cat^R^ cj0046*::*cj1110c*fdxApr* *kan^R^*

a kan^R^, kanamycin antibiotic resistance cassette; cat^R^, chloramphenicol antibiotic resistance cassette;

* denotes genetic complementation; ^fdxApr^, gene under control of the *fdxA* promoter, ^cetApr^, gene under control of the *cetA* promoter. Superscript text denotes the promoter used in the complementation cassette.

### Construction of *cetA* (*cj1190c*), *cetB* (*cj1189c*), *cetAB*, *cetC* (*cj1191c*), *cetZ* (*cj1110c*), and *cheA* (*cj0284c*) mutants

Plasmids used in this study are listed in Table S1, whereas primers used are listed in Table S2. DNA fragments of the target gene and approximately 500 bp of flanking sequence on each side was PCR amplified using Phusion DNA polymerase (New England Biolabs) using the primers detailed in Table S2. These amplified fragments were purified (QIAgen PCR purification kit), digested and ligated into pNEB193 (New England Biolabs) also digested with the corresponding restriction endonucleases (see Table S2) and transformed into chemically competent *E. coli* strain Top10 (Invitrogen). Constructs containing an insert were selected on LB agar plates containing 100 µg ml^−1^ carbenicillin and 20 µg ml^−1^ X-gal. Constructs containing an insert were confirmed by restriction digest analysis and sequencing (TGAC, Norwich, UK). To make the Δ*cetA*, Δ*cheA*, and Δ*cetC* (Δ*cj1119c*) insertional inactivation constructs, the *Bam*HI-digested kanamycin cassette from pMARKan9 [22] was inserted into the naturally occurring *Bam*HI and *Bgl*II sites in the *cetA* gene, the naturally occurring *Bam*HI site in *cheA*, or the naturally occurring *Bgl*II site of *cj1191c*. Similarly, the *Bam*HI-digested chloramphenicol cassette from pAV35 was ligated into the naturally occurring *Bgl*II site of *cj1191c*. To make the Δ*cetB* and Δ*cetAB* insertional-inactivation constructs, inverse-PCR primers (see Table S2) were used to amplify the regions flanking the target genes. These purified PCR products were digested with either *Bgl*II or *Bam*HI, purified and ligated to the *Bam*HI ends of the kanamycin cassette. To make the Δ*cetZ* (Δ*cj1110c*) insertional-inactivation construct, a chloramphenicol cassette [22] with *Bam*HI ends was ligated into the naturally occurring *Bgl*II site in *cetZ*. All ligation reactions were transformed into *E. coli* strain Top10 and positive transformants were selected for by plating on LB agar supplemented with 30 µg ml^−1^ kanamycin or 30 µg ml^−1^ chloramphenicol as appropriate. Plasmids with insert, and insert orientation, were verified with restriction digest analysis and sequencing. *C. jejuni* mutant strains were isolated after transformation of the *C. jejuni* NCTC 11168 wild-type strain with the plasmids by electroporation [23], followed by selection on plates with kanamycin, chloramphenicol or both antibiotics. Colonies were screened by PCR using primers that anneal outside of the cloned flanking regions in combination with antibiotic cassette-specific primers (Table S2).

### Construction of *cetA*, *cetB*, *cetC*, *cetZ*, *cetAB*, *cetA-cetB* chimera and *cetA-cetC* chimera complementation constructs

*C. jejuni* mutants were complemented by supplying the genes of interest *in trans* using a *cj0046* pseudogene-based delivery system [24]. To make the *cetA* complementation constructs (expressed from either the *fdxA* or native *cetA* promoter), the *cetA* gene was PCR-amplified (for primers see Table S2), digested with *Nco*I, and ligated into either pCfdxA or pC46 digested with *Esp*3I (Fermentas). To make the *cetB* complementation construct, the *cetB* gene was PCR-amplified (see Table S2), digested with *Pci*I, and ligated into pCfdxA digested with *Esp*3I. To make the *cetC* complementation construct, the *cetC* gene was PCR-amplified (see Table S2), digested with *Bsp*HI, and ligated into pCfdxA digested with *Esp*3I. To make the *cetAB* complementation construct, a PCR-fragment was generated containing *cetB* and a C-terminal region of *cetA* encompassing a unique *Bam*HI site. This PCR fragmented was digested with *Bam*HI and *Pci*I and ligated into plasmid pCASO12 (*cetA*-*fdxA*pr) sequentially digested with *Bam*HI and *Esp*3I, thus adding the *cetB* gene downstream of *cetA*. To make the *cetA*-*cetB* and *cetA*-*cetC* chimeric complementation constructs, the *cetB* or *cetC* PCR fragments, digested with either *Pci*I or *Bsp*HI, were ligated into pCASO12 (see Table S1) digested with *Esp*31. To make the *cetZ* complementation construct, the *cetZ* gene was PCR-amplified (see Table S2), digested with *Pci*I, and ligated into pKfdxA digested with *Esp*31. These complementation constructs were transformed into either the wild-type strain or the appropriate mutant background using standard electroporation methods. Complementation strains were confirmed by PCR using primers that anneal outside of the *cj0046* flanking regions in combination with gene-specific primers (see Table S2).

### Growth Curves

A 50 µl single-use glycerol stock, routinely stored at −80°C, was plated on a BAB plate with Skirrow supplements and these cells were used to inoculate fresh Brucella broth. Cultures were grown in microaerobic conditions with shaking overnight at 37°C. The overnight culture was diluted to A_600_≈0.05 (∼1×10^7^ CFU ml^−1^) in 50 ml fresh Brucella broth in a T75 filter screw cap tissue culture flask (TPP, Helena Biosciences) and grown in microaerobic conditions with shaking at 37°C. The A_600_ was monitored every 60 minutes for up to ten hours.

### Autoagglutination assays and Light Microscopy

Autoagglutination (i.e. cell clumping and sedimentation) was measured as described previously by monitoring the decrease in A_600_ following incubation in a cuvette at room temperature in air [25]. For monitoring motility using light microscopy, 10 µl of overnight culture, mounted between a glass slide and coverslip, was examined using an Eclipse 50i microscope and videos acquired using a Coolpix 4500 digital camera (Nikon).

### Tube taxis assay

Tube taxis assays were prepared by mixing 0.4 g agar with 100 ml Brucella broth, followed by autoclaving. Media was cooled to ∼50°C and 1 ml of 1% filter sterile 2,3,5-triphenyltetrazolium chloride (TTC, Sigma) was added to the media. To make Resazurin tubes, 500 µl of 0.02% resazurin was added to 100 ml Brucella broth containing 0.4 g agar prior to autoclaving. After cooling to ∼45°C, 10 ml of media was added to sterile 15 ml Falcon tubes (Corning) and allowed to set. If not used directly, tubes were stored at 4°C for up to one month. A 50 µl single-use glycerol stock was plated on a BAB plate with Skirrow supplements, grown for 24 hours, and cells resuspended in 2 ml sterile PBS. The top of the agar was inoculated with 50 µl of this cell suspension. The tubes were re-capped and incubated at 37°C in either: air, 2% O_2_/10% CO_2_/88% N_2_, 5% O_2_/10% CO_2_/85% N_2_, or in air/10% CO_2_. For culture in 2% O_2_/10% CO_2_/88% N_2_ and 5% O_2_/10% CO_2_/85% N_2_, tubes were incubated in gas jars containing a modified atmosphere supplied by an Anoxomat AN2CTS (Mart Microbiology B.V). For incubation in air/10% CO_2_, tubes were incubated in a Sanyo MCO-20AIC incubator. Tubes were photographed after 24, 40, 48, 64, and 72 hours. The dye front was measured from the top of the agar using the ImageJ software and expressed as a percentage of the wild-type strain. Each strain was tested for significance using a one-sample t-test compared to a hypothetical value of 100 (GraphPad Prism 5.01).

### Swarm plate assays

All swarm plate assays were carried out using square 10 mm^2^ petri plates (Sterilin) inoculated with wild-type and three to four test strains. Swarming in rich media was assessed using Brucella broth supplemented with 0.01% TTC and 0.4% agar (Oxoid). Energy taxis assays were modified from a previous study [18], by replacing the chemically defined medium with the cell culture media DMEM and MEMα (Life Technologies). DMEM supplemented with 0.4% (w/v) agar was autoclaved and allowed to cool to ∼50°C. This medium was supplemented with 0.01% (w/v) TTC and 5% (v/v) MEMα medium (DMEM/MEMα). This medium was poured directly into plates and allowed to set for 24 hours at room temperature prior to inoculation. Plates were inoculated with 5 µl of overnight culture, and incubated in microaerobic conditions at 37°C. For any given assay, Brucella and DMEM/MEMα plates were set-up concurrently using the same overnight culture. Following inoculation, the number of viable cells in the inoculum was assayed using standard plating methods: using 96 well plates, cells were serially diluted to 1×10^−8^ then 5 µl of each dilution was spotted onto a Brucella plate. Swarm plates were photographed using a CCD camera and gel documentation system (U:Genius, Syngene) after 24 and 48 hrs and halo area measured using ImageJ (version 1.41; National Institute of Health [http://rsbweb.nih.gov/ij/]). For each plate, halo size was expressed as a percentage of the corresponding wild-type and each strain was tested for significance using a one-sample t-test, compared to a hypothetical value of 100 (GraphPad Prism 5.01).

### Chemotaxis assays

Chemotaxis assays were adapted from [26]. A cell suspension was made by resuspending cells growing on a Skirrow plate in 2 ml sterile PBS. Two hundred µl of cell suspension was mixed with 30 µl 1% TTC (filter sterile), and 3 ml 0.4% agar/PBS and quickly mixed by hand before pouring on a sterile Brucella plate. Flame-sterilized tweezers were used to place a filter paper disc (6 mm Grade AA, Whatman) on the plate. Ten µl of either 1 M L-fucose (filter sterile) or PBS (negative control) was pipetted onto the filter discs. Plates were incubated in microaerobic conditions and halos visually inspected after 24 hours.

## Results

### The CetAB system is required for efficient swarming motility

**Figure 1:**
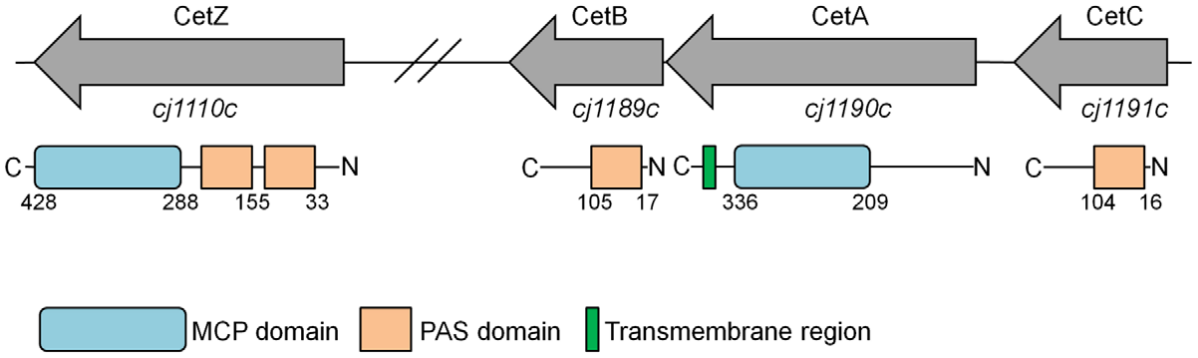
Genetic and domain organization of the *cetC*, *cetAB* and *cetZ* genes and encoded proteins in *C. jejuni* NCTC 11168, respectively. Numbers in the domain diagram show the regions of alignment according to Pfam identification.

**Figure 2:**
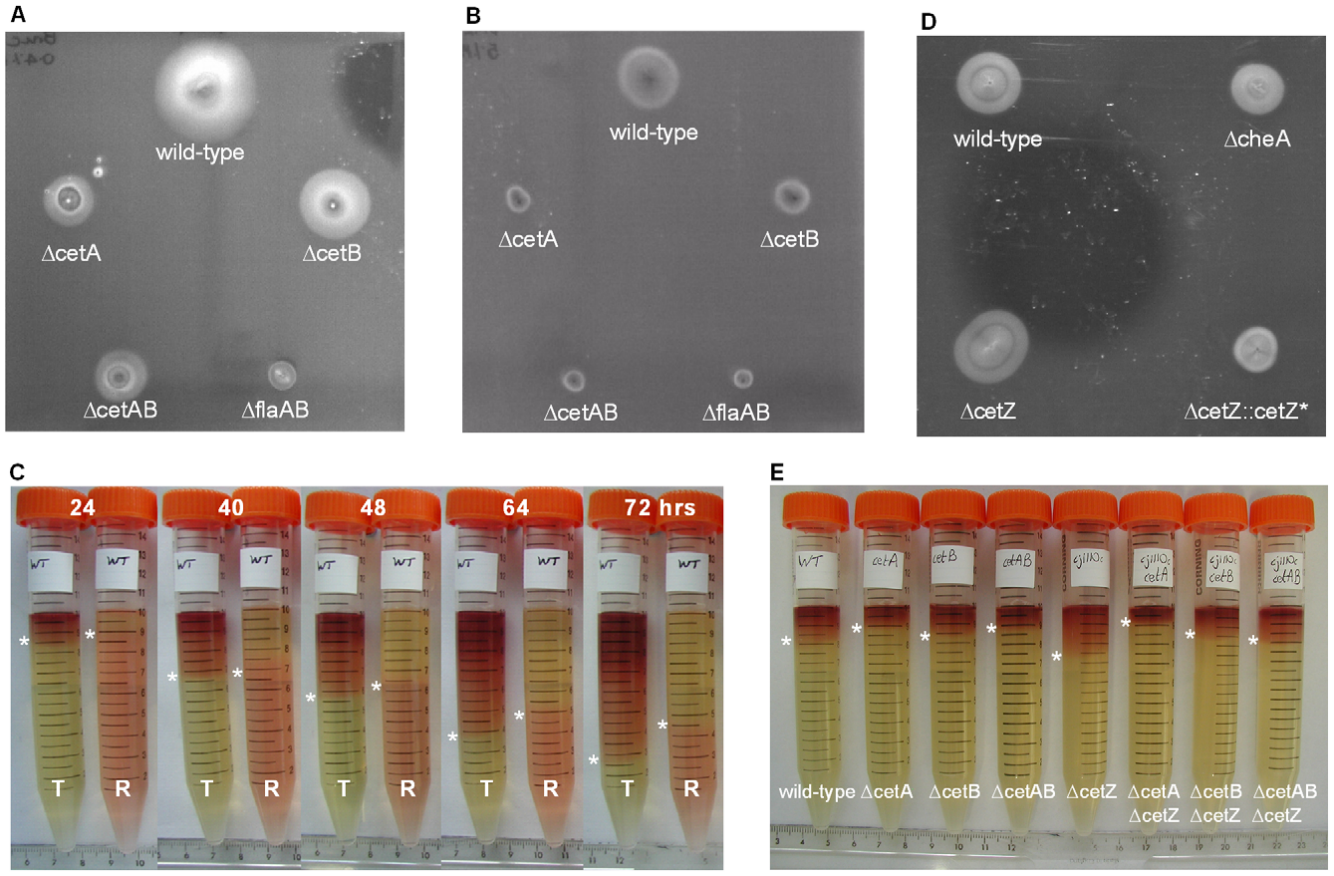
Representative swarming and taxis assays. Representative swarming plates showing wild-type, Δ*cetA*, Δ*cetB*, Δ*cetAB* and Δ*flaAB* strains after 24 hours incubation at 37°C in microaerobic conditions in: A) Brucella (0.4% agar, 0.01% TTC) and B) DMEM supplemented with 5% MEMα (0.4% agar, 0.01% TTC). C) Representative composite image of parallel TTC- and resazurin-based tube assays. Brucella soft agar was supplemented with either 0.01% TTC (T) or 0.0001% resazurin (R) and were incubated after inoculation at the top at 37°C in air and photographs taken at the times indicated. Asterisks are used to show the edge of the dye front. D) DMEM/MEMα swarming assay showing halo formation of the wild-type, Δ*cheA*, Δ*cetZ*, and Δ*cetZ*::cetZ* strains. E) Representative tube assay after 40 hrs incubation in air (37°C) showing migration of wild-type, Δ*cetA*, Δ*cetB*, Δ*cetAB*, Δ*cetZ* (*cj1110c*), Δ*cetA* Δ*cetZ*, Δ*cetB* Δ*cetZ*, and Δ*cetAB* Δ*cetZ* strains. Asterisks are used to show the edge of the dye front.

**Figure 3:**
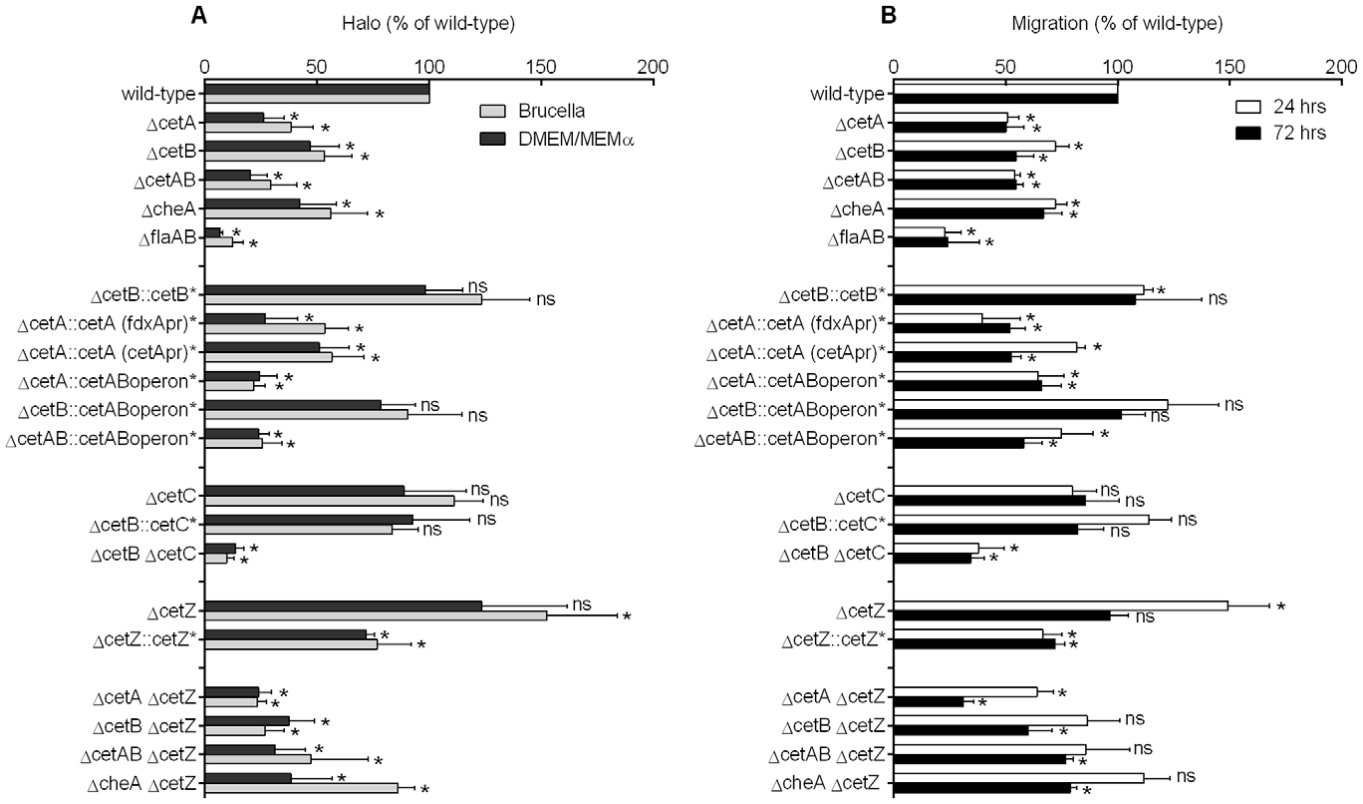
CetABC and CetZ play opposing roles in energy taxis. A) Results from swarming assays in Brucella (light grey) and DMEM/MEMα media (dark grey). B) Initial 24 hour (white) and final (black) 72 hour time points from the tube assay. Strain designations are shown on the Y-axis. All data are expressed as a percentage of the wild-type and error bars show the standard deviation from at least three biological replicates. Asterisks denote statistically significant results compared to the wild-type (one-sample t-test, p<0.05).

To investigate *cet*-dependent energy taxis in *C. jejuni*, single *cj1190c* (*cetA*), *cj1189c* (*cetB*) and double *cetAB* mutants were constructed by replacing the genes (Fig. 1) with an antibiotic cassette (see Materials and Methods). These mutants showed no growth defects in Brucella broth, either by A_600_ in Brucella broth or viable counts (data not shown). Cells exhibited normal swimming behaviour, as assessed using light microscopy, showed wild-type autoagglutination, and possessed bi-polar flagella as shown using microscopy. However, in a standard swarming motility assay in Brucella soft agar, the Δ*cetA* and Δ*cetB* mutants showed a swarming defect (40% and 50% of wild-type respectively, Fig. 2 and Fig. 3A), with the Δ*cetAB* double mutant displaying most pronounced defect (30% of wild-type, Fig. 2 and Fig. 3A). However, these phenotypes were less severe than a non-motile Δ*flaAB* mutant, where halos were 10% that of the wild-type (Fig. 2, and Fig. 3A). Assays were conducted with the wild-type strain and the Δ*cetAB* strain in the presence and absence of the redox indicator TTC (2,3,5-triphenyltetrazolium chloride [27]), which is reduced by *C. jejuni* to TPF (1,3,5-triphenylformazan), a red insoluble precipitate. No difference in the swarming phenotype was observed with or without TTC (data not shown). The presence of TTC in the agar plate aided visualisation and documentation of the halo, so all subsequent assays were performed in media supplemented with TTC at a final concentration of 0.01%.

### Redox dyes can be used to visualise and track taxis and motility of *C. jejuni*

To investigate the effect of selected *C. jejuni* mutants in energy taxis, two different assays were developed. Firstly, swarming in defined media supplemented with defined carbon sources was investigated using soft agar plates containing DMEM supplemented with 5% MEMα (DMEM/MEMα). DMEM does not support growth of *C. jejuni*, while MEMα does support growth [22]. Cells inoculated into DMEM soft-agar, supplemented with defined carbon sources (MEMα), therefore migrate away from the point of inoculation as the carbon sources are utilized, resulting in a swarming halo. Cells that lack the ability to move towards these carbon sources can be said to have a defect in energy taxis. To extend these assays, we also developed a tube-based taxis assay using 15 ml Falcon tubes, where TTC-supplemented Brucella soft agar was inoculated with a cell suspension at the top. In this assay, movement of cells is visible as a dye-front that migrates away from the point of inoculation over time (Fig. 2C). Cell migration through the agar was shown to be dependent on growth, as no migrating dye-front was observed in tubes incubated below the minimum growth temperature for *C. jejuni* (sub 35°C) or in tubes supplemented with the bacteriostatic antibiotic chloramphenicol (data not shown). Time-lapse photography was used to show that the dye-front progressed evenly over time. Migration of the dye-front was influenced by the oxygen-tension, with tubes incubated in air with 10% CO_2_ showing the greatest level of dye migration, while tubes incubated at 2% O_2_-10% CO_2_ showed the lowest level of dye migration (Fig. S1). Cell migration was independent of gravity, with the same migration observed if tubes were incubated up-side-down. All further assays were carried out at 37°C in air. A non-motile Δ*flaAB* mutant did not form halos in the DMEM/MEMα swarm plates, and this behaviour was mimicked in the tube assays, as dye migration was only 20% that compared to the wild-type strain (Fig. 2B, Fig. 3).

The benefit of using the tube assay was demonstrated when the agar was supplemented with the redox indicator resazurin (7-hydroxy-3H-phenoxazin-3-one 10-oxide) [28]. This blue dye is non-reversibly reduced to pink resorufin, then further reversibly reduced to colourless hydroresorufin [29]. Parallel assays in tubes supplemented with TTC and resazurin showed that as the cells migrated through the agar (as shown in the TTC tubes), the resazurin in the supplemented tubes was being reduced, as shown by a colour change from pink to yellow (i.e. colourless, and therefore the colour of the Brucella medium, Fig. 2C). Thus, as a result of cell growth and metabolic activity, both energy and redox gradients are established in the tubes. Comparison of swarm plates using four different strains, with and without resazurin, showed no difference in motility; hence we concluded that the presence of resazurin, like TTC, does not influence motility and cell migration (data not shown).

### The CetAB system is required for efficient energy taxis

The two taxis assays described in the previous section were used to assess whether the swarming phenotype of the Δ*cetAB* mutant was due to an energy taxis defect. The Δ*cetAB* mutant showed both poor swarming and poor migration in the tube assay (Fig. 2B, Fig. 2E, and Fig. 3). In DMEM/MEMα medium, the halo size of the *cetAB* mutant was 20% that of the wild-type, whilst in the tube assay, dye migration was 50% that of the wild-type (Fig. 3). Disrupting *cetA* and *cetB* individually also resulted in migration defects, as the Δ*cetA* strain showed 26% halo swarming and 50% migration compared to the wild-type, and the Δ*cetB* strain showed 50 to 60% swarming and migration compared to wild-type (Fig. 2E, and Fig. 3).

To show that the taxis phenotypes observed for the Δ*cetB* strain was due to the *cetB* gene, a complementation strain was constructed in which the *cetB* gene was expressed from the *C. jejuni fdxA* promoter [30]. These gene-promoter fusions were inserted into the chromosome in the pseudogene *cj0046c* [24], resulting in the Δ*cetB*::cetB* strain. Complementation of the Δ*cetB* strain by *cetB* resulted in wild-type migration in the tube assays (a small but significant increase was observed after 24 hrs in the tube assay) and wild-type halo sizes in all swarm plates suggesting that expression of CetB could rescue the migration defects observed in the Δ*cetB* mutant (Fig. 3). A complementation strain was also constructed in which the *cetA* gene was expressed from the *fdxA* promoter. Reasoning that stoichiometry between the CetA and CetB proteins may be critical for correct functioning of the sensing complex, *cetA* was also expressed from its native promoter. A strain expressing *cetA* from the *fdxA* promoter showed the same level of swarming and migration as the Δ*cetA* strain (Fig. 3). When *cetA* was expressed from its own promoter, migration was 80% of that of the wild-type strain in the early stages (24 hrs) of the tube assay (although significantly different from the wild-type) but subsequently fell to levels seen in the Δ*cetA* mutant (Fig. 3B). In the DMEM/MEMα swarming assays, this strain showed a slight increase in halo size compared to the Δ*cetA* mutant, but could not restore wild-type swarming (Fig. 3A).

Given that *cetA* and *cetB* are co-transcribed [19], disrupting *cetA* is likely to disrupt *cetB* expression. Indeed, there was no significant difference in taxis between the Δ*cetA* and Δ*cetAB* mutants in both the swarm plate and tube assays. This may account for the failure to complement a Δ*cetA* mutant with *cetA* alone. Thus, a complementation construct, containing both *cetA* and *cetB*, in the same transcriptional arrangement as the original locus, was made and transformed into the Δ*cetA*, Δ*cetB*, and Δ*cetAB* backgrounds. Strains complemented with the *cetAB* genes showed normal growth, and microscopy revealed robust swimming motility in liquid media and presence of bipolar flagella. In both the swarm plate assays and tube assays, halo size and migration of strains transformed with *cetAB* were indistinguishable from the Δ*cetA* and Δ*cetAB* mutants (Fig. 3). However, the Δ*cetB* mutant complemented with *cetAB* showed wild-type levels of swarming and migration in the tube assays. Indeed, after 24 hours in the tube assay, the Δ*cetB*::*cetAB* strain showed 120% mean migration compared to the wild-type, similar to levels seen in the Δ*cetB*::cetB* strain (Fig. 3B). This suggests that at least CetB is expressed and functional when downstream of *cetA*, and supports the findings that *cetA* and *cetB* are co-transcribed. Successful complementation with *cetB* but limited success with *cetA* could be due to the requirement for a delicate balance between the two components for a functioning sensing complex or due to technical issues with over-expression of the membrane-bound CetA. Previous attempts to complement an 81–176 Δ*cetA* strain with *cetA* were also unsuccessful [20]. Given that *cetAB* are co-transcribed and complementation with *cetA* was unsuccessful, it is not possible to fully discriminate the *cetA* and *cetAB* phenotypes. However, disruption of this locus clearly has a striking effect on taxis.

### Cj1191c (CetC) functionally complements a Δ*cetB* mutant and is required for robust taxis in concert with CetB

The genomes of all sequenced *C. jejuni* strains (except *C. jejuni* subsp. *doylei* 269.97) are annotated to contain a gene upstream of *cetA*, which encodes a protein that is 64% identical to CetB and is also predicted to encode a PAS domain protein (70% identity over the predicted PAS domain region), see Fig. 1 and Fig. S2. To determine the role of this protein in energy taxis, the *cj1191c* gene was inactivated by insertion of a kanamycin cassette. The Δ*cj1191c* strain had the same growth-rate as the wild-type strain and did not have a motility defect as shown with light microscopy and AAG. Microscopy showed normal cell morphology and bipolar flagella. The Δ*cj1191c* strain also showed wild-type levels of taxis towards L-fucose in the chemotaxis swarming assay (data not shown). In all swarm plate assays and the tube assay, halo size and migration were the same as wild-type (Fig. 3). Given the similarity to CetB, to ability of Cj1191c to complement the Δ*cetB* mutant was investigated by introducing the *cj1191c* gene, under control of the *fdxA* promoter, into the *cj0046* pseudogene in the Δ*cetB* mutant. Swarming halos and migration in the tube assay of the Δ*cetB* mutant complemented with *cj1191c* was equivalent to the wild-type showing that, like CetB, Cj1191c can also rescue the Δ*cetB* phenotype (Fig. 3). Therefore, in a Δ*cetB* mutant, a CetA-Cj1191c complex may contribute to signal transduction and taxis. To address this question, a double Δ*cetB* Δ*cj1191c* mutant was constructed by transforming the kanamycin-resistant Δ*cetB* mutant with a Δ*cj1191c::*chloramphenicol disruption construct. This double mutant had no growth defect, possessed bi-polar flagella, and showed robust swimming in liquid media. However, in the both the swarm plate and tube assays, the Δ*cetB* Δ*cj1191c* double mutant showed greatly reduced halo size (around 10% of the wild-type) and migration (Fig. 3). In Brucella swarm plates, halos from the Δ*cetB* Δ*cj1191c* mutant were significantly smaller than Δ*cetA*, Δ*cetB*, Δ*cetAB*, and Δ*cj1191c* mutants. In DMEM/MEMα swarm plates, the same trend was observed (with the exception of the Δ*cetAB* strain, Fig. 3A). In the tube assays too, the Δ*cetB* Δ*cj1191c* double mutant showed significantly less migration than the Δ*cetA*, Δ*cetB*, Δ*cetAB*, and Δ*cj1191c* mutants (Fig. 3B). Thus, while a Δ*cj1191c* mutant is not impaired for taxis, the data suggests that the Cj1191c protein can form a functional sensing complex with CetA, and that Cj1191c in concert with CetB is required for efficient energy taxis. Hence we propose to rename it CetC.

### Chimeric CetA-CetB and CetA-CetC proteins cannot rescue the Δ*cetAB* phenotype

The *cetA* and *cetB* genes are co-transcribed [19] and together are homologous to the *E. coli* aerotaxis sensor Aer. To investigate if a CetA-CetB chimeric fusion protein formed a functional sensing protein, an in-frame *cetA-cetB* fusion construct, expressed from the *fdxA* promoter, was generated. In light of the observations that both CetB and CetC were able to complement the Δ*cetB* mutant (Fig. 3), a *cetA-cetC* fusion construct was also generated. Both constructs were introduced into a Δ*cetAB* strain and swarming and tube migration assessed. In all the swarm plate assays, halos from the strains transformed with the chimeric constructs were the same as the Δ*cetAB* strain (Fig. S3, A). In the tube assays, the strains containing the chimeric constructs showed a trend towards reduced migration, even compared to the Δ*cetAB* strain (significantly different at 40 and 48 hrs, as determined by a t-test, Fig. S3, B). These strains were not impaired for growth and retained the ability to autoagglutinate. It is not clear why these fusion constructs fail to rescue the *cetAB* phenotype, but this could be due to incorrect folding of these chimeric proteins, or due to the reasons given previously for the *cetA* complementation.

### Cj1110c (CetZ) encodes a chemotaxis protein that has an opposing role to CetAB

In addition to the PAS-domain proteins encoded by *cetB* and *cetC*, the *C. jejuni* NCTC 11168 genome also contains the *cj1110c* (*tlp8*) gene, which encodes a protein containing an MCP domain and two PAS domains [7], see Fig. 1. A prediction of transmembrane regions using the DAS algorithm recommended in [31], [32], suggested that Cj1110c does not contain a recognisable transmembrane region or periplasmic export signals, and is therefore most likely located in the cytoplasm. According to the proposed classification of MCP proteins, this places Cj1110c into the IVa topology group [6], and Class IV [32].

To investigate the role of this protein in energy taxis in *C. jejuni*, the *cj1110c* was disrupted by a chloramphenicol resistance cassette. The resulting Δ*cj1110c* strain had a wild-type growth rate and did not have a motility defect, as determined using AAG and light microscopy, and had wild-type morphology including bipolar flagella. In the swarming plate assays, conversely to the phenotype shown by the *cet* mutants, halos from the Δ*cj1110c* strain were larger than those of the wild-type (Fig. 2D, and Fig. 3A). In Brucella soft-agar, the mean halo size was 150% that of the wild-type, while in DMEM/MEMα, the halo size was 120% of the wild-type (Fig. 3A). Likewise, in the TTC tube assay, migration of the dye-front of the Δ*cj1110c* strain was 150% that than the wild-type strain after 24 hours (Fig. 2E, and Fig. 3B). Complementation of the Δ*cj1110c* strain with the *cj1110c* gene expressed from the *fdxA* promoter rescued and even reversed the Δ*cj1110c* phenotype. The halo size from the swarm plate assays was 70–80% that of the wild-type (Fig. 2D, and Fig. 3A) and migration of the dye-front in the tube assay was 66% of wild-type migration after 24 hours (Fig. 3B). As all the phenotypes observed suggest an action opposite to that of CetAB, and hence we propose the name CetZ for Cj1110c.

### The *cet* phenotypes are CheA-dependent and the CetAB system predominates over CetZ

To investigate if the taxis phenotypes observed in the Δ*cetA*, Δ*cetB*, and Δ*cetZ* mutants is dependent on a functioning chemotaxis pathway, a Δ*cheA* mutant was constructed. The Δ*cheA* strain showed the same growth rate as wild-type and exhibited swimming motility as shown with light microscopy (data not shown), and microscopy confirmed the presence of bi-polar flagella. When testing for chemotactic swarming using 1 M L-fucose as a chemo-attractant, halo formation was abolished in the Δ*cheA* strain. The Δ*cetA*, Δ*cetB*, or Δ*cetAB* strains showed wild-type halo formation towards fucose suggesting that the *cet* phenotype is not due to a chemotaxis defect. The Δ*cheA* mutant showed both reduced halo size in swarming assays (Fig. 2D) and migration in tube assays (Fig. 3B), suggesting that the phenotype observed in the Δ*cetA*, Δ*cetB*, and Δ*cetAB* mutants is dependent on a functioning chemotaxis pathway.

To investigate whether CetZ uses the chemotaxis signalling pathway, a Δ*cheA* Δ*cetZ* double mutant was also made by transforming the Δ*cheA* background with the *cetZ* disruption construct. The Δ*cheA* Δ*cetZ* strain did not exhibit increased halo size or increased migration in the TTC tube assay, as observed in the Δ*cetZ* strain (Fig. 3). In DMEM/MEMα swarm plates, the halo size of the Δ*cheA* Δ*cetZ* mutant was not significantly different to the Δ*cheA* strain, whilst in Brucella media, halo size was 80% of the wild-type (Fig. 3A). In the tube assays, migration of the Δ*cheA* Δ*cetZ* strain was not significantly higher than wild-type, as the Δ*cetZ* mutant, and showed the same trend as the Δ*cheA* mutant in the later time points (Fig. 3B). The CetZ system therefore appears to be unable to bypass the CheA-mediated pathway.

If both the CetABC and CetZ systems are mediated via the chemotaxis pathway, the predominance of one system over the other was investigated by testing double mutants (Δ*cetA* Δ*cetZ*, Δ*cetB* Δ*cetZ*, Δ*cetAB* Δ*cetZ*), constructed by inactivating the *cetA*, *cetB*, and *cetAB* genes in the Δ*cetZ* background. In all the swarming assays, halo sizes were less than 50% of the wild-type (Fig. 3A) and in the tube assays, migration of the dye front was most similar to the *cet* phenotype (Fig. 2E, and Fig. 3B). These data suggest that the CetAB system predominates over the CetZ system.

## Discussion

In this work we have characterised two systems (CetABC and CetZ) that jointly govern energy taxis in *C. jejuni*, by opposing actions. Energy taxis influences movement of cells dependent on the intracellular metabolic status, and allows the cells to move towards the most favourable conditions for growth or respiration [33]. The observations that Δ*cetA* and Δ*cetB* mutants display reduced energy taxis assay in agar plates [18] have been extended by using a new, and convenient tube-based assay to track taxis as a function of the migration of a dye-front, in a column of media supplemented with a redox-indicator. The conversion of TTC in the media is a marker for metabolic activity and suggests that, as the cells grow and migrate, a local energy gradient is established as the nutrients are exhausted. Tubes supplemented with the redox indictor resazurin also show that cell growth and migration is accompanied by the formation of a redox gradient (Fig. 2C), most likely due to utilization of dissolved oxygen, the preferred respiratory electron acceptor in *C. jejuni* [34], [35]. We therefore suggest that these assays can be employed for studying energy taxis in *C. jejuni*. This simple assay may also be adaptable for studying taxis in other bacteria. The ease of set-up and data gathering make it an attractive and scalable method, especially when compared to more sophisticated but complicated assays reported, based on the use of fluorescently-labelled cells [17] or microscope video tracking of single cells [36].

The observations made with the *cetABC* and *cetZ* mutants in this study support the original hypothesis from which the *cetAB* genes derive their name [18]. Originally, Δ*cetA* and Δ*cetB* mutants in *C. jejuni* strain 81–176 showed reduced taxis towards both the carbon source sodium pyruvate and alternative electron acceptor fumarate. However, the specific effector of the CetAB system remains unknown, be it direct sensing of molecular oxygen (as in the case of HemAT in *Bacillus subtilis* [37]) or indirect redox sensing. We favour the second hypothesis, and propose that these proteins function via a redox mechanism, likely mediated through a redox-active co-factor, such as FAD, bound to the PAS domain of CetB or CetC. Given the fact that CetB is soluble in the cytoplasm, the reason of localising the CetA component in the membrane remains unknown. The topology of CetA has been delineated, with the N-terminal being cytoplasmic, and a loop region projecting into the periplasm [19]. The role of the periplasmic region in signal sensing, if any, remains an open question.

Disrupting the CetB-like protein (Cj1191c) upstream of the CetAB system alone had no discernable phenotype. However, a lack of both this CetC (Cj1191c) protein and CetB had a dramatic effect on taxis, while retaining swimming motility, suggesting that CetB and CetC may act in concert in signal transduction. Accordingly *cetC* (*cj1191c*) was observed to rescue the energy taxis phenotype of the *cetB* mutant suggesting that CetC alone can form a functioning signal transduction complex with CetA (Fig. 3). Ergo, why should the genome encode both a CetB and second ‘substitute’ PAS domain sensor? In microarray studies reporting transcriptional regulation of *cetAB*, *cetC* is not reported as being differentially regulated, whereas the *cetAB* genes display growth phase-dependent regulation of expression [38], [39], [40]. It is possible that *cetC* is constitutively expressed at a basal level such that the sensing complex can always function. Then, under appropriate stresses when the *cetAB* locus is up-regulated, CetB assumes the role of primary sensor. The fact that the Δ*cetB* energy taxis phenotype in the tube assay is less severe than that of the Δ*cetA* mutant (60% migration compared to 50% migration of wild-type) may be explained by formation of a functioning CetA - CetC complex. Moreover, this hypothesis may go someway to explain the epithelial cell invasion phenotypes described previously [20]. Only the Δ*cetA* strain, but not the Δ*cetB* strain, was shown to have an invasion defect for human epithelial cells. If signal transduction through the Cet complex is required for invasion, it is possible that in the Δ*cetB* mutant, CetC functions in place of CetB to form a functional signalling complex. The data from the tube assays containing the chimeric CetA-CetB and CetA-CetC constructs showed reduced cell migration even compared to the Δ*cetAB* mutant. However, in all experiments where complementation with *cetA* was attempted, wild-type taxis could not be restored, whether *cetA* alone (Δ*cetA::cetA* ^fdxApr^*), as a transcriptional unit with *cetB* (11168 Δ*cetA::cetAB* ^fdxApr^*) or as a chimeric construct fused to either *cetB* or *cetC*. CetA complementation therefore seems beyond the technical limitations of the genetic tools used in this study. Given that CetB and CetC are expressed as discrete proteins, the ability of the PAS domain proteins to transiently interact with CetA may be critical for signal transduction. Correct functioning of the Cet complex may require dynamic interaction between CetA and both PAS domain proteins.

**Figure 4:**
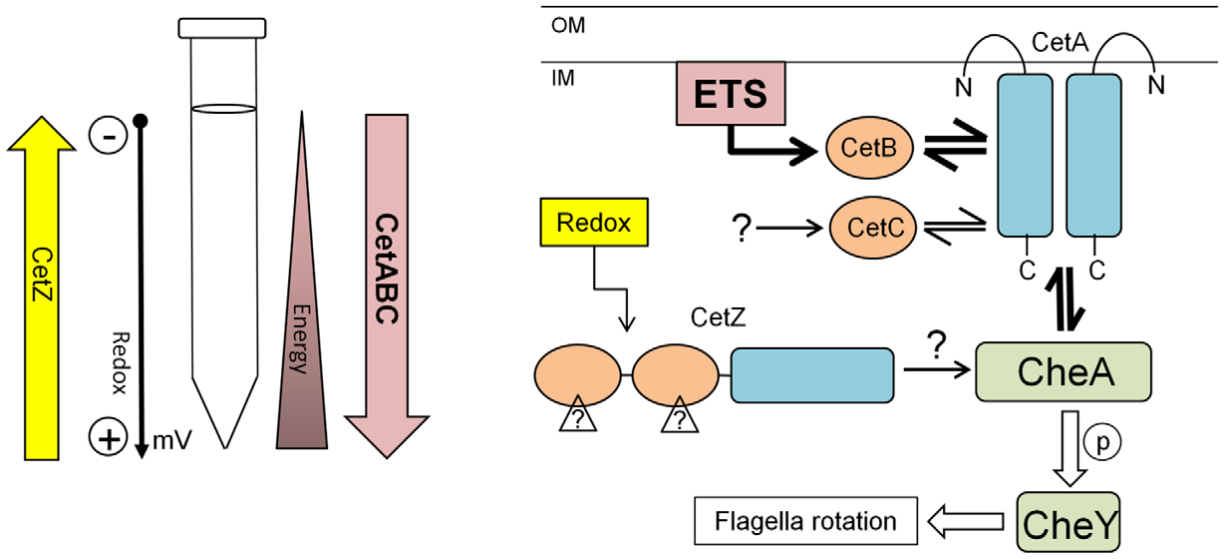
Model of how the combined activity of the CetABC and CetZ systems control energy taxis in *C. jejuni*. A) Diagram showing our assumptions about the tube taxis assay (right), schematically displaying a hypothesis on role and sensing by the CetZ and CetABC systems. B) Model displaying possible signal transduction by the CetABC and CetZ system (left, adapted from [19]) and their interaction with the chemotaxis pathway via the CheA protein. The dominant CetABC pathway is shown by bold arrows (see discussion for more information). ETS, electron transport chain; OM, outer membrane; IM, inner membrane.

The results of the energy taxis assays with the Δ*cetZ* (Δ*cj1110c*) mutant clearly demonstrated an opposing phenotype compared to that observed in the Δ*cetAB* mutants, a phenotype that could be rescued, and even reversed, by complementation (Fig. 2D, and Fig. 3). Thus, if the CetAB system has a role in energy taxis, does the opposing function of CetZ control taxis away from favourable nutritional environments? We suggest that CetZ is a redox sensor, which mediates taxis away from high redox potentials, associated with high oxygen concentrations (Fig. 4). In addition to the data presented here, the work on the role of the CetZ-ortholog AerC in *A. brasilense* is particularly revealing [11]. This protein shares the same domain architecture of CetZ and functions to mediate taxis away from high oxygen tensions (and thus redox potentials) to microaerobic conditions (low oxygen tension/redox potential) to permit nitrogen fixation by the oxygen-sensitive nitrogenase complex [11]. Given its predicted cytoplasmic location, CetZ may sense a cytoplasmic redox-active metabolite, or use a redox-sensing cofactor, such as FAD (as is the case for *E. coli* Aer, the N-terminal PAS domain of *Azotobacter vinelandii* NifL [41] and *A. brasilense* AerC [11]), although direct interaction with the respiratory chain cannot be ruled out. PAS domains are known to be involved in direct protein-protein interactions, often facilitating homo- and hetero-dimer formation [12]. NifL from *A. vinelandii* contains two tandem PAS domains, the second PAS domain lacking any bound cofactor and involved in homodimer formation [42]. In the published *C. jejuni* protein-protein interaction network [43], CetZ shows interactions with 19 other proteins, including CheV (in *C. jejuni*, a hybrid CheW-response regulator protein), but no other components of the chemotaxis pathway or respiratory complex. Thus, the precise sensing mechanism used by CetZ requires further investigation.

A double Δ*cetAB* Δ*cetZ* strain was used to demonstrate that the CetAB system pre-dominates over the CetZ system (Fig. 3), as this mutant displayed the reduced taxis of the Δ*cetAB* mutant. Thus, for *C. jejuni*, the ability to seek out an environment for optimal energy generation probably supersedes redox taxis. Indeed, energy taxis has been shown to be the primary mechanism *Campylobacter* uses to navigate to an optimal oxygen tension [33]. This perhaps reflects the ability of *C. jejuni* to utilize multiple electron acceptors with different redox potentials, ranging from the preferred electron acceptor oxygen (+820 mV) down to fumarate (+30 mV) [35]. Moreover, *C. jejuni* possesses two terminal oxidases: a lower affinity CioAB system and a high affinity *cb*-type cytochrome *c* oxidase [44], which may permit respiration at very low oxygen concentrations. It should also be noted that in *E. coli* too, the Aer system, which is most similar to the CetAB system, predominates over chemotaxis mediated by the Tsr, Tar, Trg, and Tap chemoreceptors [45].

The energy taxis phenotype of the *cheA* mutant showed 50–70% swarming/migration compared to the wild-type strain, suggesting that the CheA-CheY signal transduction pathway has an explicit role in energy taxis. However, the *cheA* phenotype was less striking than the *cetAB* phenotype, suggesting that taxis is not entirely abolished. So, while CheA is likely the primary pathway for energy taxis signal transduction, bypasses may exist, such as promiscuous phosphorylation of CheY.

In summary, we present data demonstrating that *C. jejuni* contains two taxis systems which have opposing roles, which are likely to function to maintain the cells at an optimum energy and redox balance. CetABC is an energy taxis system, utilizing two cytoplasmic sensing modules (CetB and CetC), which drives cells to favourable conditions for energy generation whilst CetZ drives cells away from high redox potentials (Fig. 4). Both pathways mediate their effects through CheA and the downstream chemotaxis system. The CetAB system predominates over CetZ, suggesting that while *C. jejuni* can fall back on sub-optimal electron acceptors for respiration, an exodus from low energy environments drives taxis permitting continued survival and proliferation.

## Supporting Information

Figure S1 **Taxis of** ***C. jejuni*** **NCTC 11168 in the TTC-based tube assay is dependent on the oxygen tension, as shown after quantification of energy taxis in different atmospheric conditions.**
(PDF)

Figure S2 **Genetic organization of the** ***cetABCZ*** **loci in the** ***Campylobacter*** **genus based on available genome sequences.** Genes are drawn roughly to scale and additional flanking genes are hashed. Pseudogenes are shown by interrupted boxes.
(PDF)

Figure S3 **Energy taxis phenotype of the** ***C. jejuni*** **Δ** ***cetAB*** **strain, and the Δ** ***cetAB*** **strain complemented with** ***cetA*** **-** ***cetB*** **and** ***cetA*** **-** ***cetC*** **chimeric constructs.**
(PDF)

Table S1 **Plasmids used in this study.**
(PDF)

Table S2 **Primers used in this study.**
(PDF)
