# Supplementary material for: Signal Balancing by the CetABC and CetZ Chemoreceptors Controls Energy Taxis in Campylobacter jejuni
Source: PLoS One. 2013 Jan 29;8(1):e54390. doi: 10.1371/journal.pone.0054390 (PMC3558505; doi:10.1371/journal.pone.0054390)
Supplement: Figure S1 — Taxis of C. jejuni NCTC 11168 in the TTC-based tube assay is dependent on the oxygen tension, as shown after quantification of energy taxis in different atmospheric conditions. (PDF) [file pone.0054390.s001.pdf]

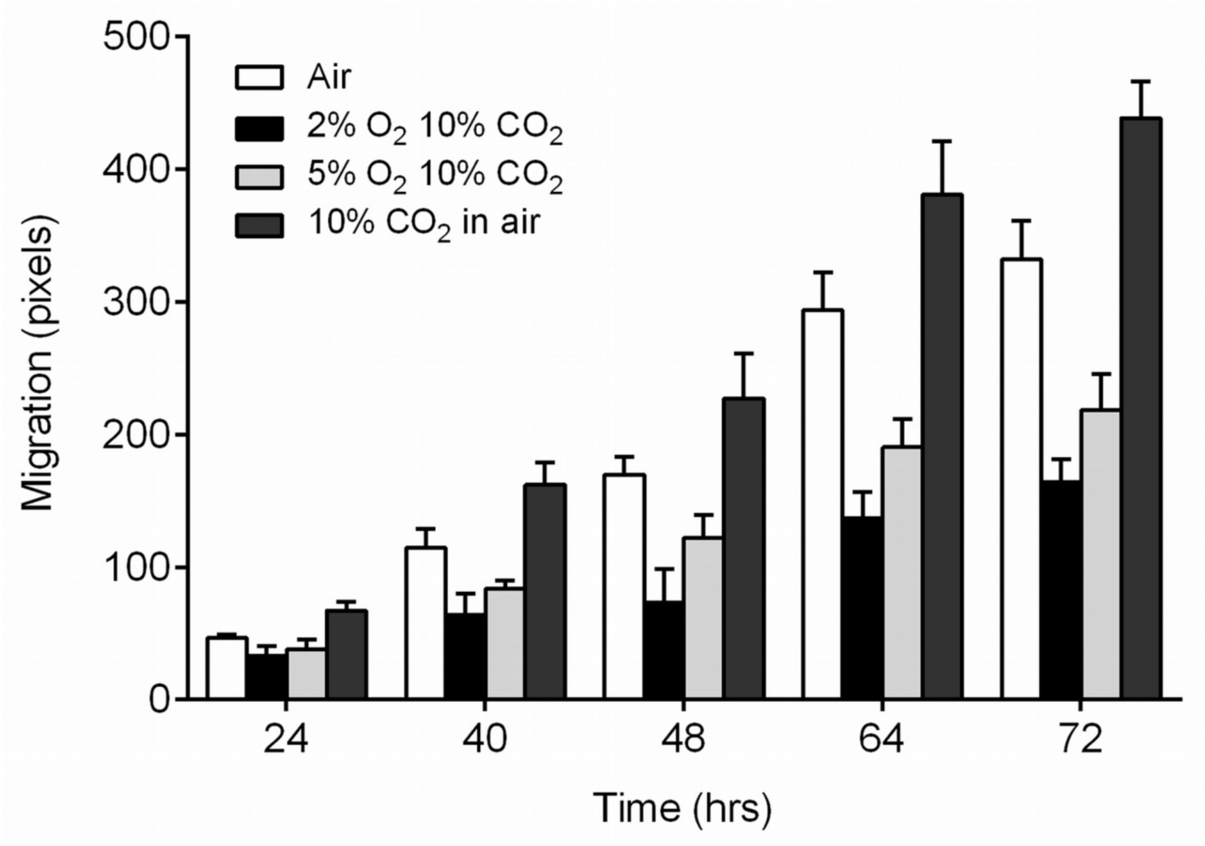

Figure S1.

Taxis in the tube assay is dependent on oxygen tension. Quantification of energy taxis in different atmospheric conditions. Tubes were incubated at 37ºC in either: air, 2% oxygen + 10% CO_2, 5% oxygen + 10% CO_2, or in air in a CO_2 incubator (10% CO_2) and photographed on five occasions from 24 to 72 hours. Dye migration was measured using ImageJ. Error bars show the standard deviation from three biological replicates.
