# Supplementary material for: Signal Balancing by the CetABC and CetZ Chemoreceptors Controls Energy Taxis in Campylobacter jejuni
Source: PLoS One. 2013 Jan 29;8(1):e54390. doi: 10.1371/journal.pone.0054390 (PMC3558505; doi:10.1371/journal.pone.0054390)
Supplement: Figure S2 — Genetic organization of the cetABCZ loci in the Campylobacter genus based on available genome sequences. Genes are drawn roughly to scale and additional flanking genes are hashed. Pseudogenes are shown by interrupted boxes. (PDF) [file pone.0054390.s002.pdf]

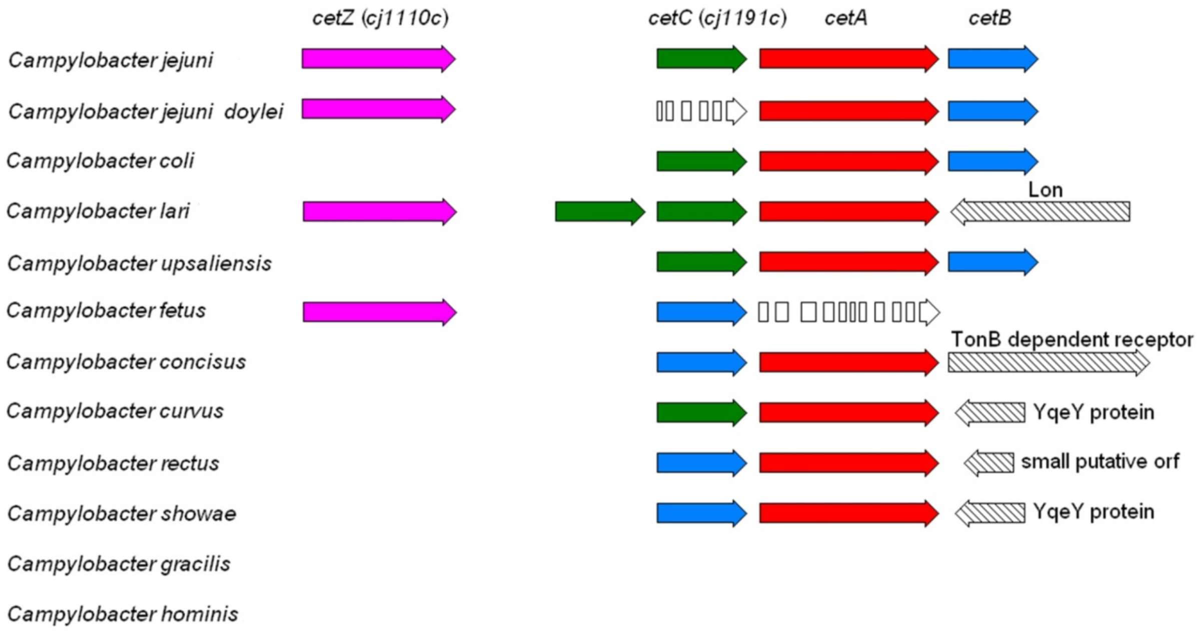

Figure S2.

Genetic organization of the *cetABCZ* loci in the *Campylobacter* genus based on available genome sequences. Genes are drawn roughly to scale and additional flanking genes are hashed. Pseudogenes are shown by interrupted boxes. The protein sequences encoded by the *cet* genes were searched against a FASTA file containing all open reading frames for each genome using a local BLAST search, using both BLASTP and TBLASTN (BioEdit - http://www.mbio.ncsu.edu/bioedit/bioedit.html). Homologs of the Cet proteins were then re-checked against the NCTC 11168 sequences to confirm the match and gene order confirmed using Artemis. In *C. jejuni* subsp. *doylei* strain 269.97, an *aer1* ortholog is present but interrupted by several stop codons and therefore considered to be a pseudogene. The *cetABC* genes are conserved in the *jejuni* species of the *Campylobacter* genus, with the exception of *C. jejuni* subsp. *doylei*, where *cj1191c* is present as a pseudogene, suggesting functional redundancy. Other species from the *Campylobacter* genus show variations of this gene arrangement: *C. coli* and *C. upsaliensis* are most similar to *C. jejuni* with similarly arranged loci. *C. concisus*, *C. curvus*, *C. rectus*, and *C. showae* have genes encoding proteins with a single PAS domain upstream of a gene encoding a CetA homolog, and no gene encoding a

CetZ homolog; while *C. lari* contains two genes encoding CetC homologs upstream of genes for CetA and a CetZ homolog. The *C. fetus* genome encodes a CetB homolog upstream of a *cetA* pseudogene, and a number of genes encoding other proteins that contain PAS domains, including a CetZ homolog (Cff8240_0322) and two proteins that contain PAS, GGDEF, and EAL domains that may be involved in cyclic di-GMP signalling [1]. A full description of chemotaxis proteins in *C. fetus* is described in [2]. *C. gracilis*, and *C. hominis* appear to lack both the *cetABC* and *cetZ* loci entirely. Interestingly, the *cet* loci are absent in the *Helicobacter* species *H. pylori, H. acinonychis*, and *H. hepaticus*. A chemotaxis protein, ChePep, has recently been identified in *H. pylori* lacking any similarity to known chemotaxis proteins except for a CheY-like receiver domain [3,4]. Moreover, *H. pylori* employs three CheV proteins, each with a distinct role in chemotaxis [5], and lacks CheR and CheB. These differences may reflect host adaptation in *H. pylori* [6,7] where both energy and pH taxis systems appear to be important for colonization of their particular niche [8,9].

References

1. Romling U, Gomelsky M, Galperin MY (2005) C-di-GMP: the dawning of a novel bacterial signalling system. Mol Microbiol 57: 629-639.
2. Fahmy D, Day CJ, Korolik V (2012) Comparative in silico analysis of chemotaxis system of *Campylobacter fetus*. Arch Microbiol 194: 57-63.
3. Howitt MR, Lee JY, Lertsethtakarn P, Vogelmann R, Joubert LM, et al. (2011) ChePep controls *Helicobacter pylori* Infection of the gastric glands and chemotaxis in the *Epsilonproteobacteria*. MBio 2: e00098-00011.
4. Sweeney EG, Guillemin K (2011) A gastric pathogen moves chemotaxis in a new direction. MBio 2: e00201-00211.
5. Lowenthal AC, Simon C, Fair AS, Mehmood K, Terry K, et al. (2009) A fixed-time diffusion analysis method determines that the three cheV genes of Helicobacter pylori differentially affect motility. Microbiology 155: 1181-1191.
6. Gilbreath JJ, Cody WL, Merrell DS, Hendrixson DR (2011) Change is good: variations in common biological mechanisms in the epsilonproteobacterial genera Campylobacter and Helicobacter. Microbiol Mol Biol Rev 75: 84-132.
7. Lertsethtakarn P, Ottemann KM, Hendrixson DR (2011) Motility and chemotaxis in *Campylobacter* and *Helicobacter*. Annu Rev Microbiol 65: 389-410.
8. Schweinitzer T, Mizote T, Ishikawa N, Dudnik A, Inatsu S, et al. (2008) Functional characterization and mutagenesis of the proposed behavioral sensor TlpD of *Helicobacter pylori*. J Bacteriol 190: 3244-3255.

9. Croxen MA, Sisson G, Melano R, Hoffman PS (2006) The Helicobacter pylori chemotaxis receptor TlpB (HP0103) is required for pH taxis and for colonization of the gastric mucosa. J Bacteriol 188: 2656-2665.
