# Supplementary material for: Signal Balancing by the CetABC and CetZ Chemoreceptors Controls Energy Taxis in Campylobacter jejuni
Source: PLoS One. 2013 Jan 29;8(1):e54390. doi: 10.1371/journal.pone.0054390 (PMC3558505; doi:10.1371/journal.pone.0054390)
Supplement: Figure S3 — Energy taxis phenotype of the C. jejuni Δ cetAB strain, and the Δ cetAB strain complemented with cetA - cetB and cetA - cetC chimeric constructs. (PDF) [file pone.0054390.s003.pdf]

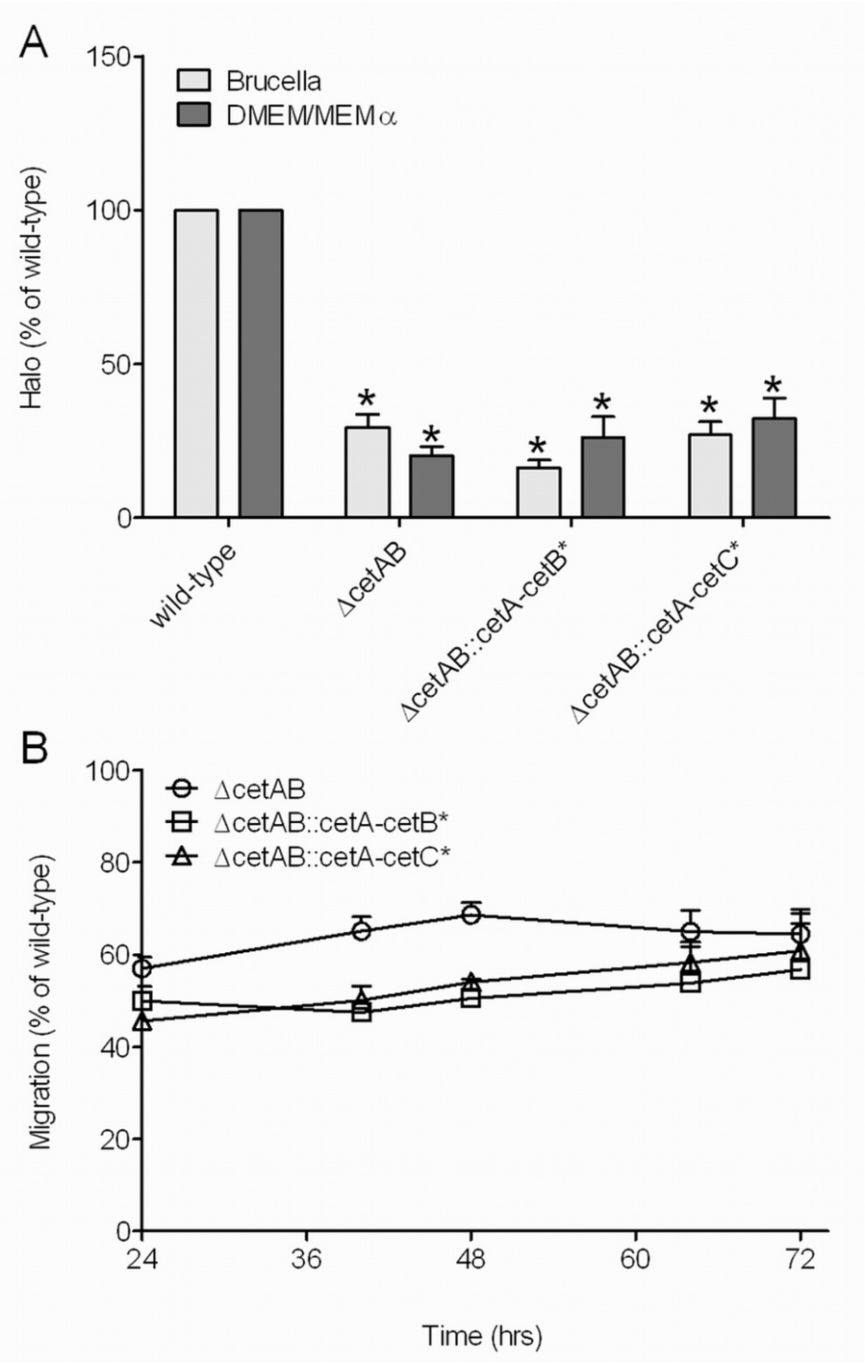

Figure S3.

Energy taxis phenotype of the Δ*cetAB* and Δ*cetAB* strain complemented with *cetA*-*cetB* and *cetA*-*cetC* chimeric constructs. A) Results from swarming assays in Brucella (light grey) and DMEM/MEMα media (dark grey). B) Time course of dye migration. Error bars show the standard deviation from three biological replicates.
