# Supplementary material for: Signal Balancing by the CetABC and CetZ Chemoreceptors Controls Energy Taxis in Campylobacter jejuni
Source: PLoS One. 2013 Jan 29;8(1):e54390. doi: 10.1371/journal.pone.0054390 (PMC3558505; doi:10.1371/journal.pone.0054390)
Supplement: Table S1 — Plasmids used in this study. (PDF) [file pone.0054390.s004.pdf]

Table S1. Plasmids used in this study.

**Plasmids**	Description [a]
pNEB193	General cloning vector
pCfdxA	Complementation plasmid containing *cj0046* flanks, Chloramphenicol resistance cassette and *fdxA* promoter
pKfdxA	Complementation plasmid containing *cj0046* flanks, Kanamycin resistance cassette and *fdxA* promoter
pC46	Complementation plasmid containing *cj0046* flanks, Chloramphenicol resistance cassette (for native promoter complementation
pCASO5	*cetA* disruption plasmid, kan^R
pCASO6	*cetAB* disruption plasmid, kan^R
pCASO8	*cetB* disruption plasmid, kan^R
pCASO10	*cj1110c* (*cetZ*) disruption plasmid, cat^R
pCASO11	*cj1191c* (*cetC*) disruption plasmid, cat^R
pCASO11Kan	*cj1191c* (*cetC*) disruption plasmid, kan^R
pCASO12	*cetA* complementation plasmid – *fdxA* promoter, cat^R
pCASO13	*cetA-cetB* chimera complementation plasmid, cat^R
pCASO14	*cetA-cetC* chimera complementation plasmid, cat^R
pCASO15	*cheA* disruption plasmid, kan^R
pCASO16	*cetB* complementation plasmid – *fdxA* promoter, cat^R
pCASO17	*cetC* complementation plasmid – *fdxA* promoter, cat^R
pCASO21	*cetA* complementation plasmid – *cetA* promoter, cat^R
pCASO22	*cetZ* complementation plasmid – *fdxA* promoter, kan^R

pCASO31	*cetAB* operon complementation plasmid – *fdxA* promoter, cat^R
pMARKan9	Source of Kanamycin resistance cassette [1]
pAV35	Source of Chloramphenicol resistance cassette [1]

a. kan^R, kanamycin resistance; cat^R, chloramphenicol resistance

Reference

1. van Vliet AHM, Wooldridge KG, Ketley JM (1998) Iron-responsive gene regulation in a *Campylobacter jejuni fur* mutant. J Bacteriol 180: 5291-5298.
