# Supplementary material for: Signal Balancing by the CetABC and CetZ Chemoreceptors Controls Energy Taxis in Campylobacter jejuni
Source: PLoS One. 2013 Jan 29;8(1):e54390. doi: 10.1371/journal.pone.0054390 (PMC3558505; doi:10.1371/journal.pone.0054390)
Supplement: Table S2 — Primers used in this study. (PDF) [file pone.0054390.s005.pdf]

Table S2. Primers used in this study.

Name	Sequence [a]	Description
CetAflankFwd	5'-GCCAATAATGAATTCTTAAAATACGCAG-3'	Upstream primer to amplify *cetAB locus* plus ≈500bp flanking sequence.
CetBflankRev	5'-GCCTTTAACTGCAGCATTTTCTACG-3'	Downstream primer to amplify *cetAB* locus plus ≈500bp flanking sequence.
CetBflankFwd	5'-CTACAAGCGAGAATTCTATTGCAATTC-3'	Upstream primer to amplify *cetB* plus ≈500bp flanking sequence.
CetAflankRev	5'-CAAACATATTCTGCAGTGAAAAATGTG-3'	Downstream primer to amplify *cetA* plus ≈500bp flanking sequence.
cj1191cflankFwd	5'-CCGCTTTAGCACGAATTCTACCTAC-3'	Upstream primer to amplify *cj1191c* plus ≈500bp flanking sequence.
cj1191cflankRev	5'-CCTTTTAAACCTGCAGGCAAAGCC-3'	Downstream primer to amplify *cj1191c* plus ≈500bp flanking sequence.
cetBStartInverseBglII-B	5'-gctaAGATCTCATTTGCATAAACTATTTTACC-3'	N-terminal inverse PCR primer for replacing *cetB* with the kanamycin cassette.
cetBEndInverseBglII-B	5'-cgatAGATCTGCAGATAAAACTTATAATGAGC-3'	C-terminal inverse PCR primer for replacing *cetB* with the kanamycin cassette.
cj1191cStartInverseBglII	5'-gctaAGATCTGGCATAAATGATATTCCCC-3'	N-terminal inverse PCR primer for replacing *cj1191c* with the kanamycin cassette.
cj1191cEndInverseBglII	5'-cgatAGATCTGGAGTTTCAGCTTTAATGG-3'	C-terminal inverse PCR primer for replacing *cj1191c* with the kanamycin cassette.
cetBStartInverse	5'-gctaGGATCCCATTTGCATAAACTATTTTACC-3'	N-terminal inverse PCR primer for replacing *cetB* and the C-terminal region of *cetA* with the kanamycin cassette.
cetBEndInverse	5'-cgatGGATCCGCAGATAAAACTTATAATGAGC-3'	C-terminal inverse PCR primer for replacing *cetB* and the C-terminal region of *cetA* with the kanamycin cassette.
KmReadOut	5'-CGGGGAAGAACAGTATGTCGAGC-3'	Reads out from the 3' end of the Kanamycin resistance cassette
KmPrReadOut	5'-GCGATATCTTCTATATAAGCGTACCG-3'	Reads out from the 5' end of the Kanamycin resistance cassette
CatReadOut	5'- CGTTTGTGACGGCTTTCATGTTTGC-3'	Reads out from the 3' end of the Chloramphenicol resistance cassette
CatPrReadOut	5'-GGTCGAAATACTCTTTTCGTGTCC-3'	Reads out from the 5' end of the Chloramphenicol resistance cassette
cetAfusion1	5'-GGGGTAAATCCATGGTGAAAAATGG-3'	Anneals to the 5' end of *cetA* for cloning into complementation vector (fdxA promoter)
cetAprFwd	5'-GGATATTGTAAGTCCATGGAAAATGAC-3'	Anneals upstream of *cetA* promoter for cloning into complementation vector (native promoter)
cetARev	5'-CCACCCCCATGGTTTATATTTTTAATTTTGC-3'	Anneals to 3' end of *cetA* (downstream of stop codon) for cloning into complementation vector
cetAfusion2	5'-CCCACCATGGCAATTGATATTTTTAATTTTGC-3'	Anneals to 3' end of *cetA* and changes the stop codon to a serine codon to allow for construction of the CetA-CetB chimeric protein
cetBfusion1	5'-GAGGGTGGGAACATGTCAAGAG-3'	Anneals to the 5' end of *cetB* for cloning into complementation vector.
cetBfusion2	5'-CGTTTTAAGTCTAACATGTATTTATTTAGC-3'	Anneals to the 3' end of *cetB* for cloning into complementation vector.
cj1191cfusion1	5'-GGATGTTTTAAATCATGAAAGAAATAG-3'	Anneals to the 5' end of *cj1191c* for cloning into complementation vector.
cj1191cfusion2	5'- GATTAAATTCATGATATTTAATTATTTTCTTG-3'	Anneals to the 3' end of *cj1191c* for cloning into complementation vector.
Cj1110cCompFwd	5'-GGTGAGTTACATGTTTGGTGCTAAG-3'	Anneals to the 5' end of *cj1110c* and

		introduces a *Pci*I site for cloning into complementation vector.
Cj1110cCompRev	5'-CGTGAAAAACATGTTTATGACATCG-3'	Anneals to the 3' end of *cj1110c* for cloning into complementation vector.
0046Fcheck3	5'-GCAGAGCACTTGATTTTAGTGTGTGC-3'	Primer for checking inserts into pseudogene *cj0046* used for complementation (upstream primer).
0046Rcheck2	5'-GCAAAAATCATCCTAAAAGATCC-3'	Primer for checking inserts into pseudogene *cj0046* used for complementation (downstream primer).
cj1190c	5'-GATCTACAAGCGAAATTTCTA-3'	Primer that anneals in *cetA* and used with cetBfusion2 for amplifying *cetB* and C-terminal portion of *cetA* for making *cetAB* operon complementation construct.

a) Restriction enzyme sites that are introduced for cloning purposes are underlined.

Stretches of mismatched bases are in lowercase.
